# Cancer Screening among Immigrants Living in Urban and Regional Australia: Results from the 45 and Up Study

**DOI:** 10.3390/ijerph110808251

**Published:** 2014-08-14

**Authors:** Marianne F. Weber, May Chiew, Eleonora Feletto, Clare Kahn, Freddy Sitas, Lucy Webster

**Affiliations:** 1 Cancer Research Division, Cancer Council NSW, P.O. Box 572 Kings Cross, NSW 1340, Australia; E-Mails: chiewm@wpro.who.int (M.C.); eleonoraf@nswcc.org.au (E.F.); clarek@nswcc.org.au (C.K.); freddys@nswcc.org.au (F.S.); 2 School of Public Health, University of Sydney, NSW 2006, Australia; 3 School of Public Health and Community Medicine, University of New South Wales, NSW 2052, Australia; 4 School of Biomedical Sciences, Charles Sturt University, Boorooma Street, Wagga Wagga, NSW 2650, Australia; E-Mail: lwebster@csu.edu.au

**Keywords:** cancer screening, mammogram, faecal occult blood test, prostate specific antigen test, immigrants, regional Australia, geographic variation

## Abstract

Over 25% of the Australian population are immigrants, and are less active participants in cancer screening programmes. Most immigrants live in urban areas of Australia, but a significant proportion (~20%), live in regional areas. This study explored differences in cancer screening participation by place of birth and residence. Self-reported use of mammogram, faecal occult blood test (FOBT), and/or prostate specific antigen (PSA) tests was obtained from 48,642 immigrants and 141,275 Australian-born participants aged 50 years or older in the 45 and Up Study (New South Wales, Australia 2006–2010). Poisson regression was used to estimate relative risks of test use, adjusting for key socio-demographic characteristics. Overall, immigrants from Asia and Europe were less likely to have had any of the tests in the previous two years than Australian-born participants. Regional Australian-born participants were more likely to have had any of the tests than those living in urban areas. Regional immigrant participants were more likely to have had an FOBT or PSA test than those living in urban areas, but there were no differences in mammograms. This report identifies key immigrant groups in urban and regional areas that policymakers and healthcare providers should target with culturally appropriate information to promote cancer screening

## 1. Introduction

Australia is a culturally diverse and geographically vast nation, with over a quarter of the population born overseas [1] and nearly one third of the population living outside the main urban centres [2]. In 2010, cancer was estimated to be responsible for the largest proportion of the total burden of disease in Australia [3], and many resources have been invested in cancer prevention and treatment [4]. Screening programmes are key initiatives for early detection of cancer and Australia has organised national screening programmes for bowel, breast, and cervical cancer [5,6,7] as well as high rates of opportunistic prostate-specific antigen (PSA) testing for prostate cancer [8].

Cancer screening can be controversial, with the number of lives saved through early diagnosis weighed against potential over-diagnosis and over-treatment of indolent tumours [9]. In the case of prostate cancer, contradictory evidence from large trials renders the continuing widespread use of PSA testing particularly complex [10]. Nevertheless, reductions in mortality rates have been proven for breast, cervical, bowel, and prostate cancer screening [11,12,13,14].

Participation rates in cancer screening programmes are not optimal across all demographic sub-groups. Even though the screening paradigm is evolving from compliance encouragement to informed consent [15], national screening programmes require significant government investment and high participation rates to effectuate their purpose. Previous Australian studies have shown variation in screening uptake between people living in urban compared to regional areas [16,17,18,19] and also among immigrant groups [5,7,20]. Although people from non-English speaking backgrounds are less active participants in cancer screening, participation by immigrants living in regional areas has not been explored.

The objective of this report was to investigate potential differences in screening for bowel, breast and prostate cancer among immigrants living in urban *vs*. regional areas of New South Wales (NSW), Australia, in the 45 and Up Study cohort [21]. The 45 and Up Study oversampled people living in regional and remote areas of NSW in order to investigate geographic variation in health in finer detail. Furthermore, in an earlier report from the 45 and Up Study, when the cohort was half its current size, we found significant differences in cancer screening uptake by place of birth [22]. With an estimated 20% of immigrants in Australia living outside a capital city [1], and with immigration policies in Australia introducing initiatives encouraging newly arrived immigrants to settle in regional areas [23], the variation in cancer screening participation among immigrants by their place of residence is of increasing importance.

## 2. Experimental Section

### 2.1. Study Population

The Sax Institute’s 45 and Up Study is a population-based cohort study of people aged 45 and over in NSW, Australia. Participants were randomly sampled from Medicare Australia, Australia’s universal health insurance system, which includes Australian citizens and permanent residents as well as some temporary residents and refugees. Residents in regional areas and those aged 80 and over were over-sampled by a factor of two, and all residents in remote areas were sampled [21].

Participants completed a self-administered health and lifestyle baseline questionnaire in English. The participation rate was 18%, however the 45 and Up Study sample has excellent heterogeneity and is reasonably representative of the NSW population, is the largest cohort study in Australia [24] and it has a response rate that is comparable to similar studies internationally [25,26,27,28]. This paper uses the baseline cross-sectional data from 232,056 participants aged 50 years and over who completed the questionnaire between January 2006 and February 2010. Participation in the national cervical cancer screening programme [6] was not included in the baseline questionnaire and is not part of this analysis. The lower age limit of 50 years was chosen because screening for bowel and breast cancer is not recommended for people younger than 50 if they are at normal risk, as is PSA testing in some Australian guidelines (e.g., [29]).

The 45 and Up Study has been approved by the University of New South Wales Human Research Ethics Committee. Additional ethics approval for this specific project was provided by the Cancer Council NSW Ethics Committee.

### 2.2. Screening History

Cancer screening was ascertained by self-report from the following questions: (1) “Have you ever been screened for colorectal (bowel) cancer? If yes, please indicate which test(s) you had”. We restricted our results to faecal occult blood test (FOBT) use rather than colonoscopy or sigmoidoscopy because the National Bowel Cancer Screening Program uses FOBTs, and colonoscopy/sigmoidoscopy can be used as a diagnostic test as well as a screening test; (2) “Have you ever been for a breast screening mammogram?”; (3) “Have you ever had a blood test ordered by your doctor to check for prostate disease? (PSA test)”. We were not able to distinguish men who had a PSA test for prostate cancer screening from those who may have had a PSA test to investigate disease. However, approximately two thirds of the PSA tests administered Australia-wide in 2008 were for cancer screening [30]. For all tests, respondents were asked to indicate how long ago (in years) they had used each test type and this analysis focussed on tests received in the previous two years.

### 2.3. Place of Birth and Place of Residence

Self-reported place of birth was grouped according to an aggregated version of that used in the Global Burden of Disease Study (see Table 1) [31]. Place of residence was grouped according to the Accessibility/Remoteness Index of Australia (ARIA+; 2001 [32]) into five standard categories and were then collapsed into either urban (major city: ARIA 1) or regional locations (inner regional, outer regional, remote, very remote: ARIA 2–5) [32].

### 2.4. Analysis

We examined the proportion of immigrants and Australian-born participants who reported having a screening test in the previous two years in urban *vs*. regional areas. Poisson regression models with robust standard errors were used to derive relative risks (RR) and 95% confidence intervals (CI) of screening by place of residence (urban/regional ARIA+) and place of birth, using appropriate interaction terms [33]. To account for the oversampling of participants aged 80 years and older and those living in regional and remote areas, sampling weights were used in regression analyses with weights equal to the inverse probability of selection into the study. Each test type was analysed separately and, for FOBT, stratified by sex.

All models were adjusted for age (in single years), family history of any cancer type (bowel/breast/prostate, other, none), education (none, 10 years of schooling, 12 years of schooling or trade/diploma, and university degree), annual household income from all sources (<$5k, $5k–$9k, $10k–$19k, $20k–$29k, $30k–$39k, $40k–$49k, $50k–$69k, >$70k), private health insurance (yes/no), relationship status (single/partner), partially or fully retired (yes/no), and for mammograms, ever used hormone replacement therapy (HRT; yes/no).

## 3. Results and Discussion

Of the 232,056 participants in the cohort aged 50 years and older, 39,897 (17.2%) reported ever having cancer (excluding non-melanoma skin cancer) and were excluded from analysis to avoid overestimation of cancer screening in this high-risk population. An additional 2083 participants (0.9%) who did not specify a place of birth and 159 participants (0.07%) who did not have an ARIA score for their place of residence were also excluded.

The distribution of key socio-demographic characteristics by place of birth and residence is shown in Table 1. Of the 189,917 included participants, 45.1% lived in an urban area and 54.9% lived in a regional area. After adjusting for potential confounders, there was a significant interaction between place of birth and place of residence for FOBT use among men (*p* = 0.005) but not women (*p* = 0.08). There was also a significant interaction between place of birth and place of residence for mammogram uptake among women (*p* = 0.004), however the interaction between place of birth and place of residence for PSA testing among men was not significant (*p* = 0.76).

Among all men in the cohort, 20,127 (22.72%) had a FOBT and 51,943 (58.64%) had a PSA test in the previous two years. Among women, 18,541 (18.30%) had a FOBT and 69,156 (68.24%) had a mammogram in the previous two years. Table 2 shows that after adjusting for key demographic variables, Australian-born participants living in regional areas were more likely to have had a FOBT in the previous two years than Australian-born participants living in urban areas. There was little variation in PSA test and mammogram use by place of residence among Australian-born participants. A similar pattern was observed for immigrant participants, with those living in regional areas more likely to have had a FOBT than their urban counterparts. Immigrants living in regional areas were slightly (6%) more likely to have had a PSA test than urban immigrants, but there was no difference in mammogram uptake by place of residence.

**Figure 1:**
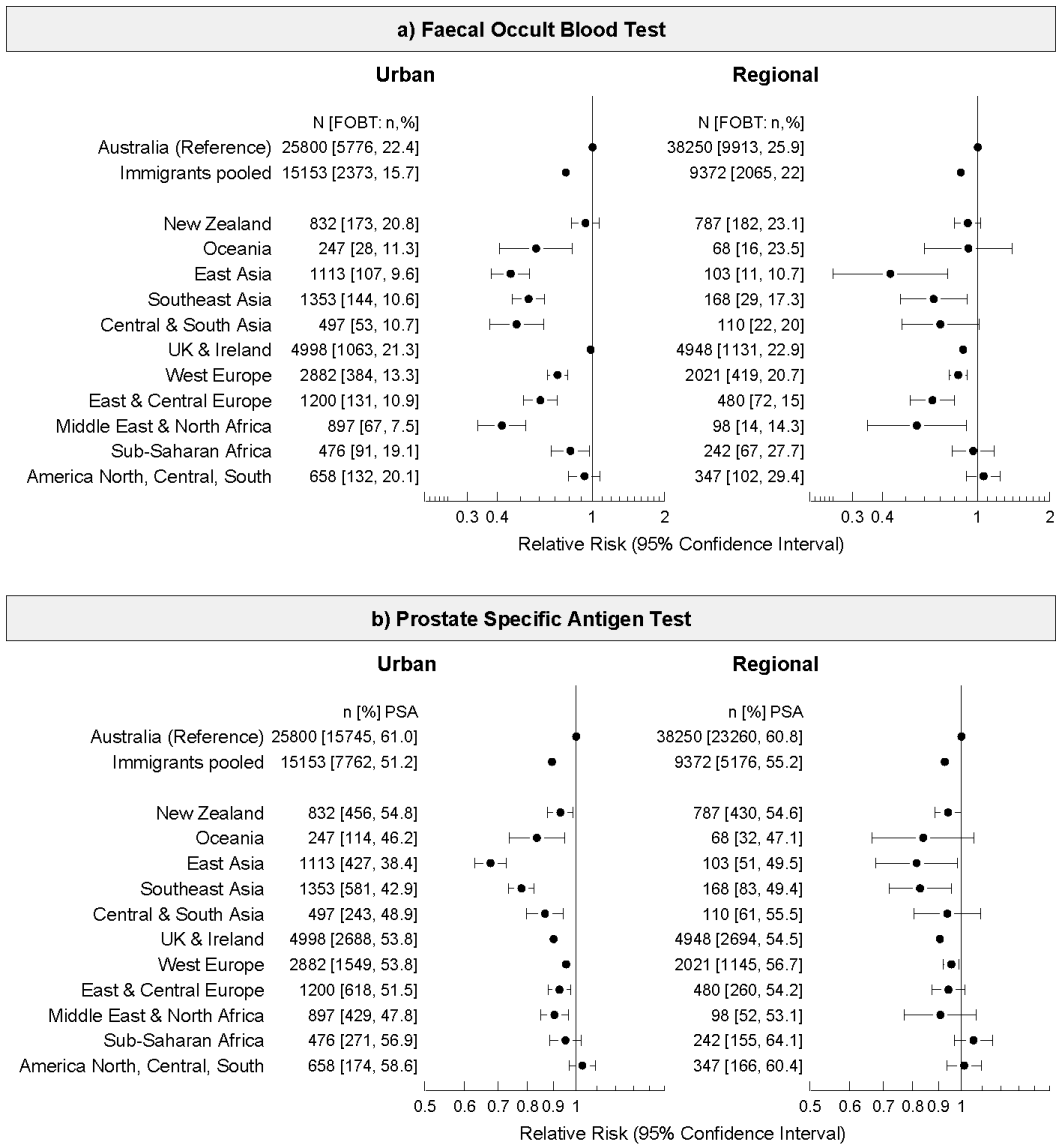
Relative Risk and 95% confidence intervals of (**a**) faecal occult blood test (FOBT), and (**b**) prostate specific antigen (PSA) test use, in the last two years among men living in urban and regional areas of New South Wales, Australia by place of birth in the 45 and Up Study (2006–2010). Relative risks adjusted for age, family history of any cancer type, education, income, health insurance status, relationship status, and retirement status.

**Figure 2:**
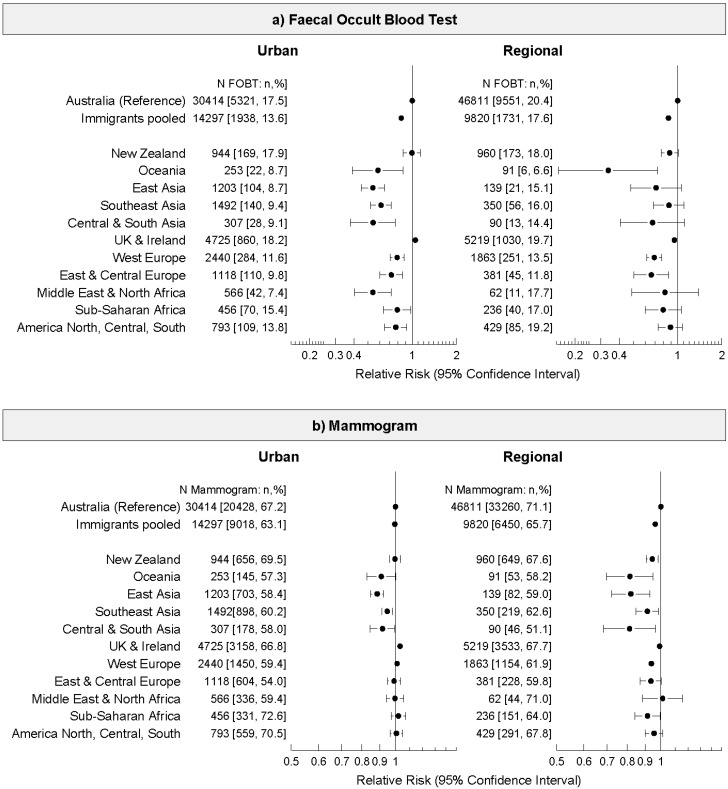
Relative risks and 95% confidence intervals of (**a**) faecal occult blood test (FOBT), and (**b**) mammogram use, in the last two years among women living in urban and regional areas of New South Wales, Australia by place of birth in the 45 and Up Study (2006–2010). Relative risks adjusted for age, family history of any cancer type, education, income, health insurance status, relationship status, retirement status, and hormone replacement therapy.

To explore these differences further, we analysed cancer screening uptake by place of birth for urban and regional areas separately (Figure 1 and Figure 2). In both urban and regional areas, immigrants (pooled) had lower FOBT use in the previous two years than those born in Australia (men urban RR 0.78, 95% CI 0.74–0.81; men regional RR 0.85, 0.82–0.89; and women urban RR 0.84 95% CI 0.80–0.88; women regional OR 0.87, 95% CI 0.83–0.91) and lower PSA test use (urban RR 0.90, 95% CI 0.88–0.91; regional RR 0.93, 95% CI 0.91–0.94). There was very little variation in mammogram uptake in urban areas by place of birth, however in regional areas immigrants (pooled) were slightly less likely to have had a mammogram (RR 0.96, 95% CI 0.95–0.98).

Among men living in urban areas, immigrants from all places of birth, except New Zealand, North, South and Central America, and UK and Ireland, were less likely to have had a FOBT than Australian-born men (Figure 1a). In regional areas, men born in East Asia, Southeast Asia, UK and Ireland, West Europe, East and Central Europe, and North Africa and the Middle East were less likely to have had a FOBT than Australian born men (Figure 1a). PSA test use also varied by place of birth, particularly for men living in urban areas (Figure 1b) where men born in East Asia were 32% less likely to have been tested (RR 0.68, 95% CI 0.63–0.73).

Among women living in urban areas, immigrants from all places of birth, except New Zealand, and the UK and Ireland, were significantly less likely to have had a FOBT than Australian-born women (Figure 2a). In regional areas, only women from Oceania (RR 0.34, 95% CI 0.16–0.73), West Europe (RR 0.69, 95% CI 0.62–0.78) and East and Central Europe (RR 0.66, 95% CI 0.50–0.87) were significantly less likely to have had a FOBT than Australian-born women.

Figure 2b shows that in urban areas, only women born in all Asian regions were significantly less likely to have a mammogram than Australian-born women. A similar pattern of results was observed for women in regional areas, with the addition that women born in New Zealand, Oceania, and West Europe were also less likely to have had a mammogram than Australian-born women.

Perceived disparities in the health of Australians have long been a concern in healthcare and have been addressed with cancer specific initiatives such as the National Cancer Workforce Strategic Framework [34], which highlight the need for access to care by place of residence and indicate the growing importance of cultural diversity. The 45 and Up Study provides useful information on these possible disparities. In this analysis we found that both immigrants and Australian-born participants living in regional areas were more likely to have had a FOBT and a PSA test than their urban counterparts. For mammogram use, there were no differences between immigrant women pooled by place of residence, but among Australian-born women, those living in regional areas were significantly more likely to screen than those in urban areas.

**Table 1:** Sociodemographic characteristics of the study population according to place of birth (45 and Up Study 2006–2010)

Place of Birth	Urban Resident (*n* = 85,664)						Regional Resident (*n* = 104,253)					
	n	Male (%)	Age (Mean [SD], Years)	University Degree (%)	Income * ≥ 70,000 p.a. (%)	Private Health Insurance (%)	n	Male (%)	Age (Mean [SD], Years)	University Degree (%)	Income * ≥ 70,000 p.a. (%)	Private Health Insurance (%)
Australia	56,214	45.9	64.7 [10.8]	26.2	28.5	61.6	85,061	45.0	63.4 [9.4]	17.6	17.9	46.7
New Zealand	1776	46.9	62.2 [9.8]	30.0	38.3	54.1	1747	45.1	62.0 [9.0]	21.2	21.9	37.3
Oceania	500	49.4	61.7 [9.4]	18.0	17.4	40.2	159	42.8	60.3 [9.4]	28.3	28.9	47.8
East Asia	2316	48.1	62.0 [10.4]	41.5	17.0	52.3	242	42.6	62.0 [10.0]	35.5	17.4	50.4
Southeast Asia	2845	47.6	61.8 [10.0]	38.5	18.2	41.6	518	32.4	61.8 [9.6]	40.7	16.4	37.5
Central & South Asia	804	61.8	63.7 [10.5]	58.8	28.1	51.7	200	55.0	65.6 [9.6]	40.5	22.5	47.0
UK & Ireland	9723	51.4	66.0 [10.8]	27.1	28.6	53.4	10,167	48.7	65.6 [9.4]	20.7	16.4	40.4
West Europe	5322	54.2	67.7 [10.7]	16.2	15.4	44.1	3884	52.0	65.9 [9.4]	14.5	11.2	36.2
East & Central Europe	2318	51.8	68.5 [11.4]	21.4	12.9	40.4	861	55.8	67.0 [9.7]	16.0	8.5	29.4
Middle East & North Africa	1463	61.3	63.6 [10.0]	25.3	10.8	33.1	160	61.3	63.8 [9.4]	32.5	23.1	42.5
Sub-Saharan Africa	932	51.1	62.0 [10.1]	49.6	43.2	65.3	478	50.6	61.9 [8.8]	40.8	28.9	49.0
America North, Central & South	1451	45.4	61.9[8.8]	42.3	27.9	50.6	776	44.7	62.6 [8.7]	54.6	23.3	47.8
Immigrants pooled	29,450	51.5	65.0 [10.8]	29.2	23.0	48.5	19,192	48.8	65.0 [9.5]	22.2	16.3	39.5

* Annual household income from all sources.

**Table 2:** Relative risks (RR) and 95% confidence intervals of faecal occult blood test (FOBT), and prostate specific antigen (PSA) test, and mammography use, in the last two years among immigrants and Australian-born participants by place of residence, in the 45 and Up Study (2006–2010).

Test Type	Australian-Born				Immigrant			
	n Tested	% Tested	RR ^1^	95% CI	n Tested	% Tested	RR ^1^	95% CI
**Men**								
**FOBT**								
Urban resident	5776	22.39	1		2373	15.66	1	
Regional resident	9913	25.92	1.23	[1.20–1.27]	2065	22.03	1.42	[1.35–1.50]
**PSA Test**								
Urban resident	15,745	61.03	1		7762	51.22	1	
Regional resident	23,260	60.81	1.01	[1.00–1.03]	5176	55.23	1.06	[1.04–1.09]
**Women**								
**FOBT**								
Urban resident	5321	17.50	1		1938	13.56	1	
Regional resident	9551	20.40	1.18	[1.15–1.22]	1731	17.63	1.24	[1.17–1.32]
**Mammogram**								
Urban resident	20,428	67.17	1		9018	63.08	1	
Regional resident	33,260	71.05	1.02	[1.01–1.03]	6450	65.68	0.99	[0.97–1.00]

^1^ Relative risks adjusted for age, family history of any cancer type, education, income, health insurance status, relationship status, retirement status; and for women, hormone replacement therapy.

It is difficult to compare our findings to existing research, because previous Australian studies describing differences by place of residence in screening uptake have had mixed results. One ecological study showed that PSA testing was more prevalent in urban areas of Australia than in regional areas [19], while the opposite has been reported for bowel cancer screening [17]. For mammograms, existing results are even more varied [18,35,36,37]. The differences in study types, population, and time periods under investigation in each of these studies may account for the inconsistent findings. We found that across all groups the use of FOBT was lower than PSA testing and mammogram use, which could be due to the delivery method of the screening test. Bowel cancer screening, using FOBT kits sent to eligible participants’ homes, has been a free national programme in Australia since 2006 [38] (with a staged rollout in progress with full implementation scheduled for 2020 [39]). Prior to this, FOBT kits were available via “Bowelscan”, which is a Rotary Club initiative aiming to increase community knowledge of bowel cancer and its symptoms, and are still available outside of the national programme [40,41]. For breast cancer, the “BreastScreen” national programme has offered free mammograms to all women aged 50–69 via invitation since 1992, and has been widely advocated [42]. In contrast, PSA testing for prostate cancer has been used in Australia since the early 1990s even though it is not currently recommended as a population-based screening tool [43]. The PSA test is subsidised by the government, has had considerable media attention, and is an easy test to administer via a GP [44,45].

To investigate screening use in more detail we analysed test use by immigrant groups across urban and regional places of residence. This showed that although all immigrants were significantly less likely to have participated in screening in the previous two years than their Australian-born counterparts, after adjusting for socio-demographic factors there was less of a disparity in regional areas. This is perhaps due to increased promotion of screening availability in regional areas and/or a greater amount of community strength and engagement in these areas. Limited research has shown that community strength is higher in regional Australia than in urban areas [46], and higher levels of social integration are positively related to cancer screening participation [47].

It is also possible that immigrants in regional areas participate in cancer screening more than urban immigrants because they have generally been in Australia longer and because they are less likely to be part of the larger, more socially contained cultural enclaves of the city where cancer screening may not be the norm [48,49]. There is a documented trend of immigrants’ cancer risk reaching that of the Australian-born population over time [50] and it is particularly interesting that there is also evidence that screening behaviour itself ‘acculturates’ [51,52], including screening in this cohort [22]. Nevertheless, the immigrants living in regional areas in our study were still less likely to be screened than their Australian-born neighbours, even though they were more likely to be screened than their urban counterparts.

There are a number of factors that may influence intention to screen among immigrants. The first is the potential for a perception of low cancer risk in immigrant populations due to low incidence and mortality rates in their native countries [53]. Indeed, the mortality rates for colorectal, breast and prostate cancers are largely lower in the Australian immigrant population than the Australian-born population [50,54], which possibly reflects the “healthy immigrant effect” [55]. That is, there may be cultural differences in key exposures (e.g., diet, alcohol) which reduce the overall risk of these cancers and outweigh the risk of low screening rates in these groups. In addition, a study in NSW reported that general practitioners (GPs) of different nationalities had varying attitudes to bowel cancer screening, and that GPs overall were less likely to recommend screening to immigrants [56]. Immigrant groups with lower screening rates come from countries where cancer screening is not highly diffuse or where there is an expectation that health professionals are responsible for recommending any test use [57,58,59,60,61,62]. Additionally, immigrants were found to have poorer knowledge of bowel cancer and screening tests, lesser intent to participate in screening practises and received fewer screening recommendations from GPs than Australian-born people [63]. Targeted information on screening programmes may be used to educate under-screened populations and raise awareness of early detection, especially in urban areas.

The representativeness of our study may be an important limitation in the interpretation of our results. Cohort study participants tend to be healthier and more health conscious than non-participants [64], and considering the relatively low study participation rate (18%), the prevalence of screening in the 45 and Up Study cohort is possibly higher than in the general population. However, like most long-term cohort studies, the 45 and Up Study is designed to provide sufficient heterogeneity for valid comparisons within the cohort, rather than specific estimates of prevalence of exposure in the population. Furthermore, potential bias resulting from the “healthy cohort” effect, if it is present, and the availability of the questionnaire in only English, may have led to conservative results. Nevertheless, 45 and Up Study findings are not dissimilar to the Population Health Survey, which found that in 2010 the odds ratio of having a mammogram in the previous two years was 0.69 (95% CI 0.56–0.85) for immigrant women compared to Australian-born women, while the corresponding odds ratio in the 45 and Up Study was 0.81 (95% CI 0.77–0.85) [24].

## 4. Conclusions

Health differences between immigrants and those born in Australia as they relate to cancer is an issue gaining momentum and importance [65]. Better use of funded and implemented programmes, such as cancer screening, could be supported with evidence to guide their improvement. This report identifies key immigrant groups in urban and regional areas that policymakers and healthcare providers could target with culturally appropriate information to promote cancer screening.

## References

[1] Australian Bureau of Statistics (2006). A Picture of the Nation: The Statistician’s Report on the 2006 Census.

[2] Australian Institute of Health and Welfare (2007). Rural, Regional and Remote Health: A Study on Mortality.

[3] Begg S., Vos T., Barker B., Stevenson C., Stanley L., Lopez A. (2007). The Burden of Disease and Injury in Australia 2003.

[4] (2011). Cancer Australia. The Cancer Australia Strategic Plan 2011–2014.

[5] Australian Institute of Health and Welfare (2012). BreastScreen Australia Monitoring Report 2009–2010.

[6] Australian Institute of Health and Welfare (2013). Cervical Screening in Australia 2010–2011.

[7] Australian Institute of Health and Welfare (2013). National Bowel Cancer Screening Program Monitoring Report: July 2011–June 2012.

[8] Ranasinghe W.K., Kim S.P., Lawrentschuk N., Sengupta S., Hounsome L., Barber J., Jones R., Davis P., Bolton D., Persad R. (2014). Population-based analysis of prostate-specific antigen (PSA) screening in younger men (<55 years) in Australia. BJU Int..

[9] Barratt A.L. (2006). Cancer screening—Benefits, harms and making an informed choice. Aust. Fam. Physician.

[10] Andriole G.L., Crawford E.D., Grubb R.L., Buys S.S., Chia D., Church T.R., Fouad M.N., Isaacs C., Kvale P.A., Reding D.J. (2012). Prostate cancer screening in the randomized Prostate, Lung, Colorectal, and Ovarian Cancer Screening Trial: Mortality results after 13 years of follow-up. J. Natl. Cancer Inst..

[11] Gotzsche P.C., Nielsen M. (2011). Screening for breast cancer with mammography. Database Syst. Rev..

[12] Hewitson P., Glasziou P., Irwig L., Towler B., Watson E. (2007). Screening for colorectal cancer using the faecal occult blood test, Hemoccult. Cochrane Database Syst. Rev..

[13] Schroder F.H., Hugosson J., Carlsson S., Tammela T., Maattanen L., Auvinen A., Kwiatkowski M., Recker F., Roobol M.J. (2012). Screening for prostate cancer decreases the risk of developing metastatic disease: Findings from the European Randomized Study of Screening for Prostate Cancer (ERSPC). Eur. Urol..

[14] Canfell K., Sitas F., Beral V. (2006). Cervical cancer in Australia and the United Kingdom: Comparison of screening policy and uptake, and cancer incidence and mortality. Med. J. Aust..

[15] Stefanek M.E. (2011). Uninformed compliance or informed choice? A needed shift in our approach to cancer screening. J. Natl. Cancer Inst..

[16] Martini A., Javanparast S., Ward P.R., Baratiny G., Gill T., Cole S., Tsourtos G., Aylward P., Jiwa M., Misan G. (2011). Colorectal cancer screening in rural and remote areas: Analysis of the National Bowel Cancer Screening Program data for South Australia. Rural. Remote Health.

[17] Ward P.R., Javanparast S., Matt M.A., Martini A., Tsourtos G., Cole S., Gill T., Aylward P., Baratiny G., Jiwa M. (2011). Equity of colorectal cancer screening: Cross-sectional analysis of National Bowel Cancer Screening Program data for South Australia. Aust. N. Z. J. Public Health.

[18] Siahpush M., Singh G.K. (2002). Sociodemographic variations in breast cancer screening behavior among Australian women: Results from the 1995 National Health Survey. Prev. Med..

[19] Baade P.D., Youlden D.R., Coory M.D., Gardiner R.A., Chambers S.K. (2011). Urban-rural differences in prostate cancer outcomes in Australia: What has changed?. Med. J. Aust..

[20] Australian Institute of Health and Welfare (2008). Cervical Screening in Australia 2005–2006.

[21] Banks E., Redman S., Jorm L., Armstrong B., Bauman A., Beard J., Beral V., Byles J., Corbett S., Cumming R. (2008). Cohort profile: The 45 and Up Study. Int. J. Epidemiol..

[22] Weber M.F., Banks E., Smith D.P., O’Connell D., Sitas F. (2009). Cancer screening among migrants in an Australian cohort; cross-sectional analyses from the 45 and Up Study. BMC Public Health.

[23] Department of Immigration and Border Protection Fact Sheet 26—State Specific Regional Migration. www.immi.gov.au/media/fact-sheets/26state.htm.

[24] Mealing N.M., Banks E., Jorm L.R., Steel D.G., Clements M.S., Rogers K.D. (2010). Investigation of relative risk estimates from studies of the same population with contrasting response rates and designs. BMC Med. Res. Methodol..

[25] Nohr E.A., Frydenberg M., Henriksen T.B., Olsen J. (2006). Does low participation in cohort studies induce bias?. Epidemiology.

[26] Watts G. (2007). UK Biobank gets 10% response rate as it starts recruiting volunteers. BMJ.

[27] Day N., Oakes S., Luben R., Khaw K.T., Bingham S., Welch A., Wareham N. (1999). EPIC-Norfolk: Study design and characteristics of the cohort. European Prospective Investigation of Cancer. Br. J. Cancer.

[28] Lee C., Dobson A.J., Brown W.J., Bryson L., Byles J., Warner-Smith P., Young A.F. (2005). Cohort profile: The Australian longitudinal study on women’s health. Int. J. Epidemiol..

[29] The Urological Society of Australia and New Zealand USANZ PSA Testing Policy (2009). www.usanz.org.au/uploads/65337/ufiles/PDF/psa-testing.pdf.

[30] Medicare Australia Medical Benefits Schedule Statistics. www.medicareaustralia.gov.au/provider/medicare/mbs.jsp#N1003F.

[31] Harvard University, Institute for Health Metrics and Evaluation at the University of Washington, John Hopkins University, University of Queensland, World Health Organisation The Global Burden of Diseases, Injuries, and Risk Factors Operations Manual (Final Draft), 2008. www.globalburden.com.au/docs/final+GBD+operations+manual%202008.pdf.

[32] Australian Institute of Health and Welfare (2004). Rural, Regional and Remote Health: A Guide to Remoteness Classifications.

[33] Zou G. (2004). A modified poisson regression approach to prospective studies with binary data. Am. J. Epidemiol..

[34] Health Workforce Australia (2013). National Cancer Workforce Strategic Framework.

[35] Mitchell K.J., Fritschi L., Reid A., McEvoy S.P., Ingram D.M., Jamrozik K., Clayforth C., Byrne M.J. (2006). Rural-urban differences in the presentation, management and survival of breast cancer in Western Australia. Breast.

[36] Wilkinson D., Cameron K. (2004). Cancer and cancer risk in South Australia: What evidence for a rural-urban health differential?. Aust. J. Rural Health.

[37] Australian Institute of Health and Welfare (2008). BreastScreen Australia Monitoring Report 2004–2005.

[38] Australian Institute of Health and Welfare, Australian Government Department of Health and Ageing (2010). National Bowel Cancer Screening Program: Annual Monitoring Report 2009.

[39] Australian Government Department of Health and Ageing National Bowel Cancer Screening Program. www.cancerscreening.gov.au/internet/screening/publishing.nsf/Content/bowel-about.

[40] National Bowelscan. www.nationalbowelscan.org.au/.

[41] Cancer Council Victoria Faecal Occult Blood Tests (FOBTs). www.cancervic.org.au/preventing-cancer/attend-screening/bowel_cancer_screening/faecal_occult_blood_tests.

[42] Slaytor E.K., Ward J.E. (1998). How risks of breast cancer and benefits of screening are communicated to women: Analysis of 58 pamphlets. BMJ.

[43] Prostate Cancer Foundation of Australia Testing and Diagnosis. www.prostate.org.au/articleLive/pages/Testing-and-Diagnosis.html.

[44] Meyer F., Fradet Y. (1998). Prostate cancer: 4. Screening. Can. Med. Assoc. J..

[45] Chapman S., Barratt A., Stockler M. (2010). Let Sleeping Dogs Lie? What Men Should Know Before Getting Tested for Prostate Cancer.

[46] Adams D., Hess M. Measuring Community Engagement. https://publications.qld.gov.au/storage/f/2014-02-03T23%3A20%3A58.852Z/hess-michael-final.pdf.

[47] Ye J., Williams S.D., Xu Z. (2009). The association between social networks and colorectal cancer screening in American males and females: Data from the 2005 Health Information National Trends Survey. Cancer Causes Control.

[48] Hugo G.J. (1995). Understanding Where Immigrants Live.

[49] Schwartz S.J., Montgomery M.J., Briones E. (2006). The role of identity in acculturation among immigrant people: Theoretical propositions, empirical questions, and applied recommendations. Hum. Dev..

[50] Supramaniam R., O’Connell D.L., Tracey E.A., Sitas F. (2006). Cancer Incidence in New South Wales Migrants 1991–2001.

[51] Brown W.M., Consedine N.S., Magai C. (2006). Time spent in the United States and breast cancer screening behaviors among ethnically diverse immigrant women: Evidence for acculturation?. J. Immigr. Minor. Health.

[52] Maxwell A.E., Bastani R., Warda U.S. (2000). Demographic predictors of cancer screening among Filipino and Korean immigrants in the United States. Am. J. Prev. Med..

[53] Christou A., Katzenellenbogen J.M., Thompson S.C. (2010). Australia’s national bowel cancer screening program: Does it work for indigenous Australians?. BMC Public Health.

[54] Anikeeva O., Bi P., Hiller J.E., Ryan P., Roder D., Han G.S. (2011). Trends in cancer mortality rates among migrants in Australia: 1981–2007. Cancer Epidemiol..

[55] Flores G., Brotanek J. (2005). The healthy immigrant effect: A greater understanding might help us improve the health of all children. Arch. Pediatr. Adolesc. Med..

[56] Koo J.H., You M.Y., Liu K., Athureliya M.D., Tang C.W., Redmond D.M., Connor S.J., Leong R.W. (2012). Colorectal cancer screening practise is influenced by ethnicity of medical practitioner and patient. J. Gastroenterol. Hepatol..

[57] Donnelly T.T., Hwang J. (2013). Breast cancer screening interventions for Arabic Women: A literature review. J. Immigr. Minor. Health.

[58] Kwok C., White K., Roydhouse J.K. (2011). Chinese-Australian women’s knowledge, facilitators and barriers related to cervical cancer screening: A qualitative study. J. Immigr. Minor. Health.

[59] Ogunsiji O., Wilkes L., Peters K., Jackson D. (2013). Knowledge, attitudes and usage of cancer screening among West African migrant women. J. Clin. Nurs..

[60] Andreeva V.A., Pokhrel P. (2013). Breast cancer screening utilization among Eastern European immigrant women worldwide: A systematic literature review and a focus on psychosocial barriers. Psychooncology.

[61] Schoueri-Mychasiw N., Campbell S., Mai V. (2013). Increasing screening mammography among immigrant and minority women in Canada: A review of past interventions. J. Immigr. Minor. Health.

[62] Team V., Manderson L.H., Markovic M. (2013). From state care to self-care: Cancer screening behaviours among Russian-speaking Australian women. Aust. J. Prim. Health.

[63] Koo J.H., Arasaratnam M.M., Liu K., Redmond D.M., Connor S.J., Sung J.J., Leong R.W. (2010). Knowledge, perception and practices of colorectal cancer screening in an ethnically diverse population. Cancer Epidemiol..

[64] Breslow N.E., Day N.E. (1987). The Design and Analysis of Cohort Studies. Statistical Methods in Cancer Research.

[65] Sitas F., Gibberd A., Kahn C., Weber M.F., Chiew M., Supramaniam R., Velentzis L., Nickson C., Smith D.P., O’Connell D. (2013). Cancer incidence and mortality in people aged less than 75 years: Changes in Australia over the period 1987–2007. Cancer Epidemiol..

